# Association of deletion polymorphism rs10573247 in the HMGA2 gene with the risk of breast cancer: bioinformatic and experimental analyses

**DOI:** 10.1186/s12957-024-03415-4

**Published:** 2024-05-28

**Authors:** Kolsoom Najibi, Mehdi Moghanibashi, Sirous Naeimi

**Affiliations:** 1 Department of Biology, Faculty of Basic Sciences, Kazerun Branch, Islamic Azad University, Kazerun, Iran; 2 Department of Genetics, Faculty of Medicine, Kazerun Branch, Islamic Azad University, P.O. Box: 73135-168, Kazerun, Iran

**Keywords:** Breast Cancer, HMGA2, Deletion polymorphism rs10573247, Bioinformatic, miR-3125, miR-4476

## Abstract

**Background:**

The high mobility group A2 (HMGA2) gene is expressed extensively during early embryonic development but is inactivated in adulthood, and it is also reactivated in various benign and malignant tumors, including breast cancer. We first assessed the potential functional significance of the unstudied deletion polymorphism rs10573247 at the 3′UTR of HMGA2 on miRNA binding using bioinformatic tools, and subsequently, the association between this polymorphism and breast cancer susceptibility was investigated.

**Materials and methods:**

We applied the RNAhybrid tool to predict the functional effects of polymorphism rs10573247 located within the 3’ UTR of the HMGA2 gene on miRNA binding. Then, following DNA extraction, 141 breast cancer patients and 123 healthy controls were genotyped for polymorphism rs10573247 using RFLP-PCR with the restriction enzyme Eam1104I.

**Results:**

Our bioinformatic data have shown that polymorphism rs10573247 is located in the region that serves as a potential target site for eight miRNAs binding. Among them, miR-3125 exhibited decreased binding affinity for the allele delTT (MFE = -21.8) when compared to the allele TT (MFE = -23.9), but miR-4476 increased binding affinity for the allele delTT (MFE = -22.4) compared to the allele TT (MFE = -22.2). In addition, our results showed that the genotype TT/delTT (*p* = 0.005) and the genotype delTT/delTT (*p* = 0.029) were significantly associated with an increased risk of developing breast cancer compared to the genotype TT/TT using RFLP-PCR.

**Discussion and Conclusion:**

Our findings suggest that polymorphism rs10573247 may contribute to the risk of breast cancer through the functional effect of this polymorphism on miRNA binding.

**Supplementary Information:**

The online version contains supplementary material available at 10.1186/s12957-024-03415-4.

## Introduction

High Mobility Group A2 (HMGA2), a member of the HMGA gene family, is located on human chromosome 12q13–15 and is composed of five exons [1, 2]. It encodes for a small non-histone protein that is less than 12 kDa in size, consists of 108 amino acids, and can bind to AT-rich regions of DNA [1, 2].

HMGA2 interacts with numerous proteins, such as retinoblastoma protein (Rb) and E2 promoter-binding factor 1 (E2F1), and contributes to the formation of enhanceosomes, which in turn directly or indirectly control gene expression [3, 4].

HMGA2 is expressed extensively during early embryonic development, but in adulthood, it is primarily expressed in the liver, kidney, and uterus; however, it is also reactivated in various benign and malignant tumors [5–7], including breast cancer [8, 9].

In breast cancer, the promoter of HMGA2 is demethylated in TNBC tumors [10] or overexpressed in breast cancer tissues [9], leading to an increase at the mRNA and protein levels of HMGA2, which in turn plays a crucial role in promoting cell proliferation and conferring stem cell-like features.

Importantly, the HMGA2 mRNA possesses a lengthy untranslated region (UTR) of approximately 3000 nucleotides, making it susceptible to regulation by multiple microRNAs (miRNAs) [1]. More recent investigations have identified post-transcriptional level of HMGA2 during oncogenesis as several miRNAs that can decrease HMGA2 expression by targeting the HMGA2 3′UTR [1]. For instance, upregulation of miRNA-150 and miRNA-98 can negatively regulate HMGA2, which is beneficial for suppressing tumor metastasis [11, 12]. Moreover, the upregulation of Raf kinase inhibitory protein (RKIP) can induce the upregulation of miRNA-185, which subsequently suppresses HMGA2 expression, resulting in the inhibition of breast cancer cell growth and invasion [13]. Interestingly, a novel mechanism for the activation of HMGA2 involves chromosomal translocations that result in the loss of the HMGA2 3′UTR, leading to downregulation of HMGA2 by miRNAs [14]. Thus, 3′UTR of HMGA2 mRNA is important in gene regulation by miRNAs [15–18].

Given this, polymorphisms in the 3′UTR of HMGA2 can alter the miRNA binding sites, affecting the stability and translation of the mRNA. While several studies have assessed different polymorphism of the HMGA2 gene in various cancers, there is no report about breast cancer. Therefore, in this study, using bioinformatic tools, we first assessed the potential functional significance of deletion polymorphism rs10573247 at the 3′UTR of HMGA2 on miRNAs binding, and subsequently, the association between this polymorphism and breast cancer susceptibility was investigated in a case-control study.

## Materials and methods

### Bioinformatic analysis

We used the RNAhybrid tool (http://bibiserv.techfak.unibielefeld.de/rnahybrid) to predict the functional effects of polymorphism rs10573247 located within the 3’ UTR of the HMGA2 gene on miRNAs binding based on minimum free energy (MFE) and base pairing.

We used online tool Enrichr-KG (https://maayanlab.cloud/enrichr-kg) to extract the signaling pathways and gene ontologies associated with the HMGA2 gene. Additionally, we used the STRING database to look into protein-protein interactions that are direct (physical) and indirect (functional) bind to HMGA2.

### Sample collection

We recruited 141 breast cancer patients (51.4 ± 11.18 years) were diagnosed Namazi Hospital (Shiraz, Iran) and 123 (51.39 ± 10.85 years) healthy controls. The study received ethical approval from the ethics committee of Islamic Azad University, Kazerun Branch, and all participants willingly provided written informed consent.

### DNA extraction and genotyping

Genomic DNA was extracted from whole blood using GTP kit (gene transfer Pioneer, IRAN). The selected deletion polymorphism within the HMGA2 3′UTR region (rs10573247) was genotyped using restriction fragment length polymorphism-polymerase chain reaction (RFLP-PCR). The genomic region containing the target polymorphism was initially amplified using a forward primer, 5′- CACTTGTCAGCCTCAGAGCA-3′, and a reverse primer, 5′- ATGGTGAACTCAACCGAAG-3′. The PCR was carried out in a 20 µl reaction mixture comprising 10 ng of target DNA, 1X buffer (15 mM Tris-HCl, pH 8.0, 50 mM KCl), 1.5 mM MgCl2, 5 pM of each primer, 200 µM dNTPs, and 1 U of Taq polymerase. The amplification conditions included an initial denaturation at 95 °C for 5 min, followed by 30 cycles of amplification at 95 °C for 40 s, 65.1 °C for 35 s, and 72 °C for 1 min, and a final extension at 72 °C for 5 min. Subsequently, the PCR products were digested with the restriction enzyme Eam1104I (EarI) (Fermentas) at 37 °C for 5 h, following the manufacturer’s protocol. The digested products were separated by gel electrophoresis (1.5%), and the genotypes were determined based on the resulting band patterns.

### Statistical analysis

We performed statistical analysis using SPSS software (version 25.0). The Hardy-Weinberg equilibrium (HWE) was assessed in controls and patients using the chi-square test. The association between the polymorphism genotypes and breast cancer susceptibility was evaluated using odds ratios (ORs), and 95% confidence intervals (CIs) were calculated using logistic regression analysis. A p-value < 0.05 was considered statistically significant.

## Results

**Fig. 1:**
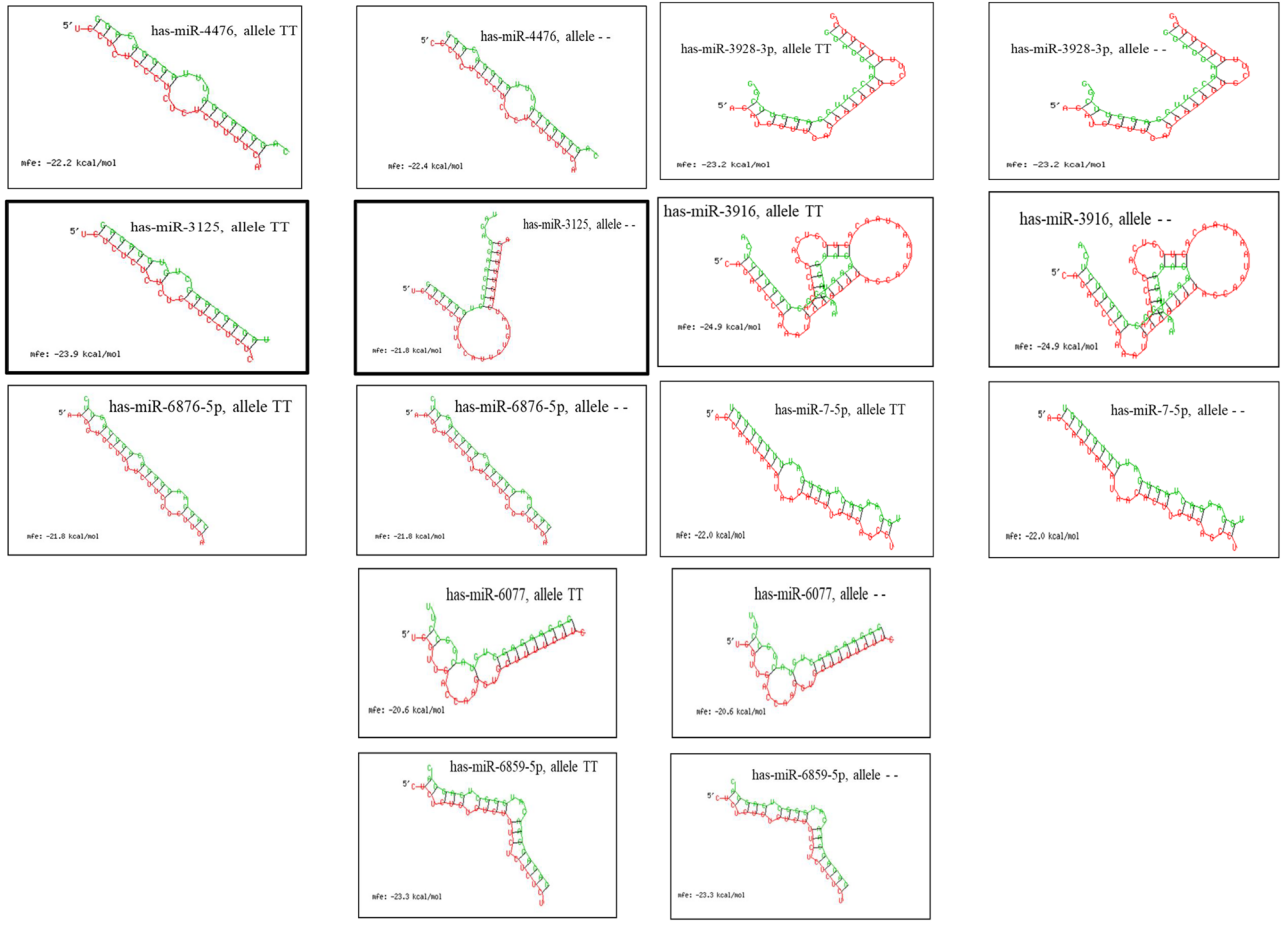
Prediction of miRNA binding sites in the 3’ UTR of HMGA2 encompassing polymorphismrs10573247 by the RNA hybrid online tool. A darker boxed line indicates the stronger influence of rs10573247 on the binding site for miR-3125 relative to other miRNAs

Using the RNA hybrid algorithm, we found that polymorphism rs10573247 at the 3’ UTR of HMGA2 serves as a potential target site for eight miRNAs (Fig. 1). While some miRNAs displayed no change in binding affinity with this polymorphism, miR-3125 exhibited decreased binding affinity for the allele delTT (MFE = -21.8) when compared to the allele TT (MFE = -23.9), indicating that the allele delTT results in weak binding to miR-3125 (delta MFE = -2.1 < 0), leading to increased expression of HMGA2 when present. Conversely, miR-4476 demonstrated increased binding affinity for the allele delTT (MFE = -22.4) compared to the allele TT (MFE = -22.2). Although the difference in MFE was slight (delta MFE = 0.2 > 0), miR-4476 exhibited increased binding affinity for the allele delTT (MFE = -22.4) compared to the allele TT (MFE = -22.2), which could potentially result in the downregulation of HMGA2.

**Fig. 2:**
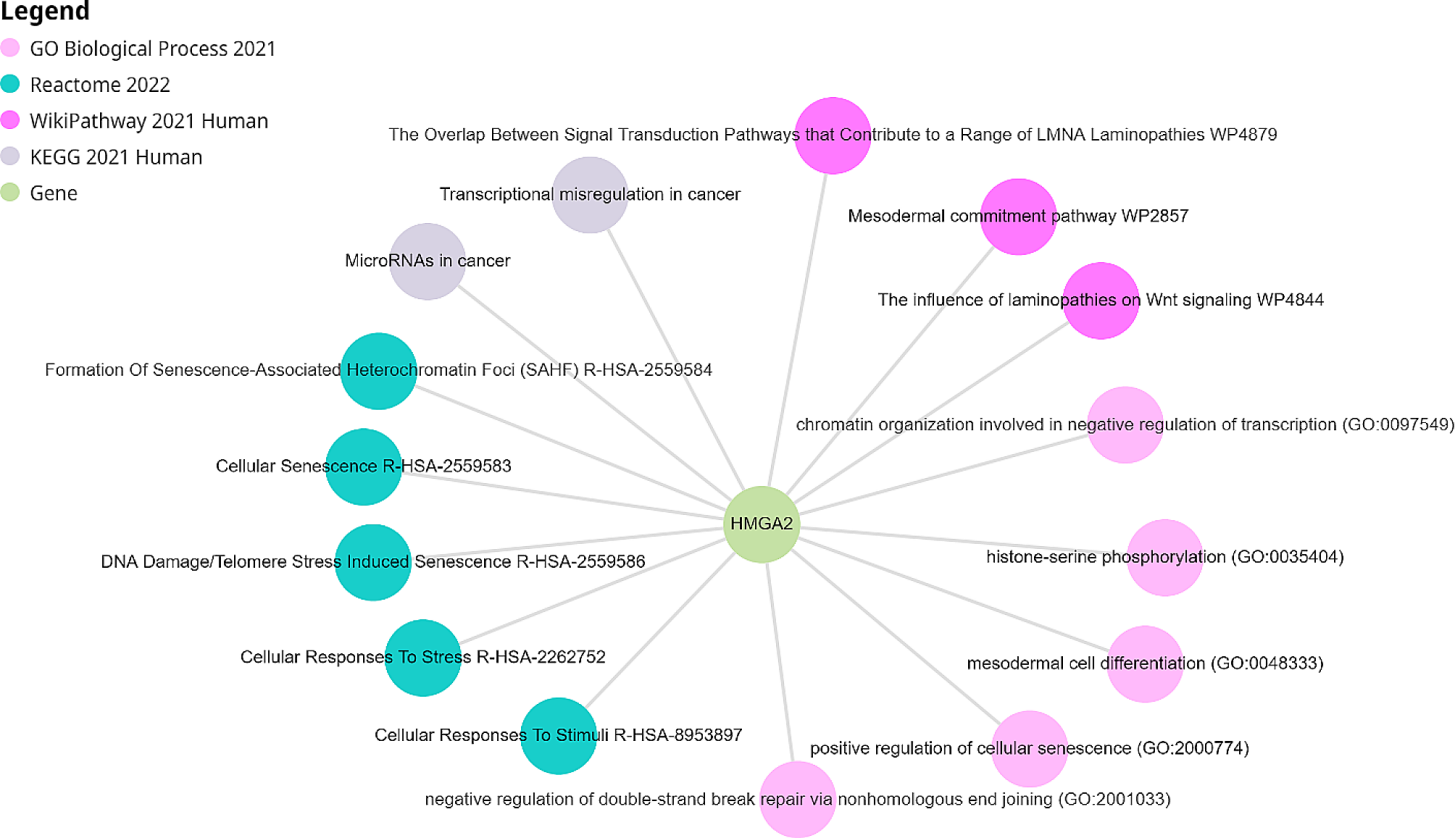
Enrichment analyses of HMGA2 gene

**Fig. 3:**
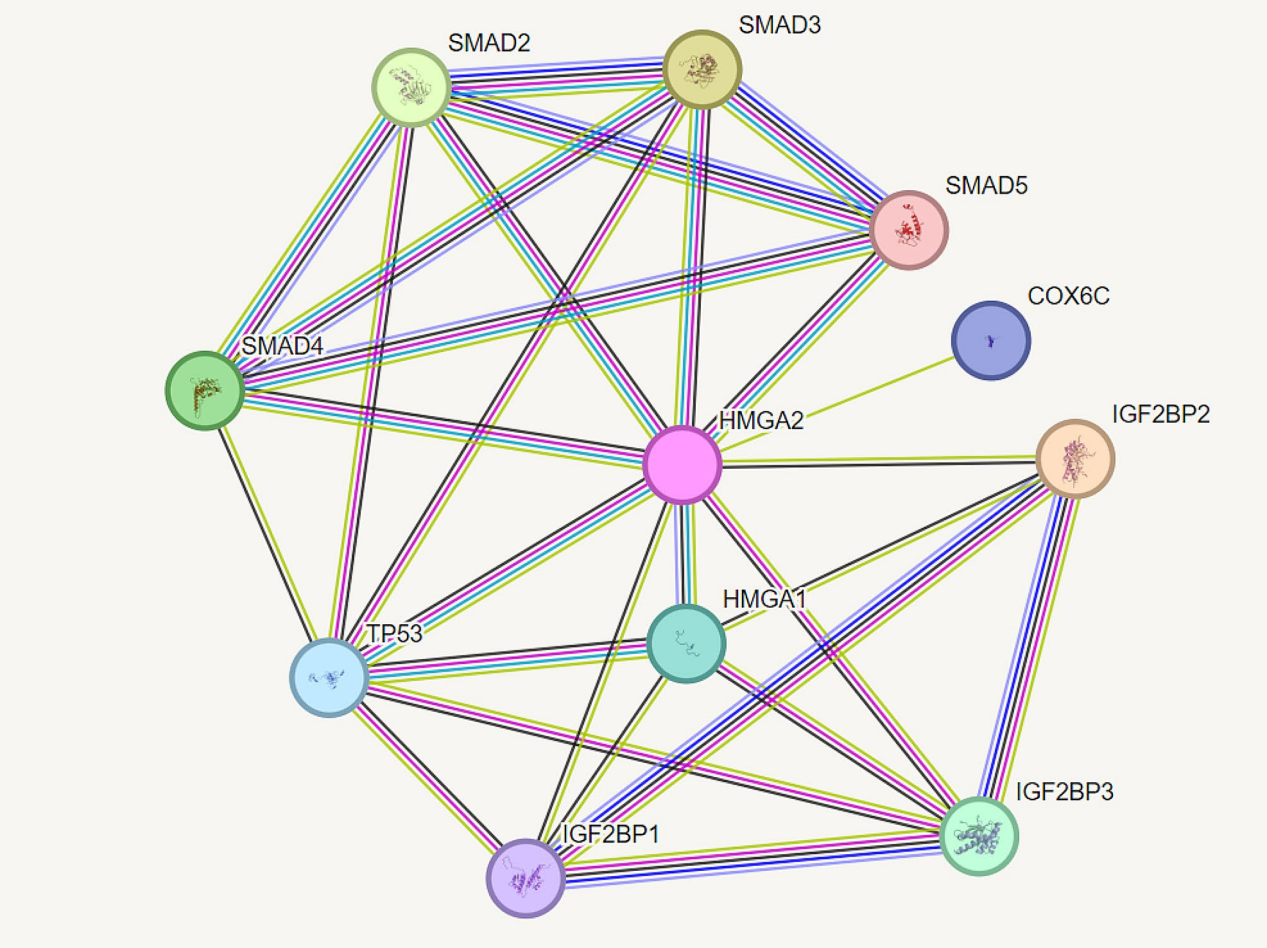
Protein-protein interactions of HMGA2 gene using STRING

GO, KEGG, Reactome and Wikipathway analyses using Enrichr have revealed that HMGA2 play a role in the process including cellular senescence, DNA repair, differentiation and chromatin remodeling and importantly in miRNA and cancers (Fig. 2). In addition, analysis in the STRING database have showed that HMGA2 interact with SMAD family, IGF2BP family and P53 proteins (Fig. 3).

**Fig. 4:**
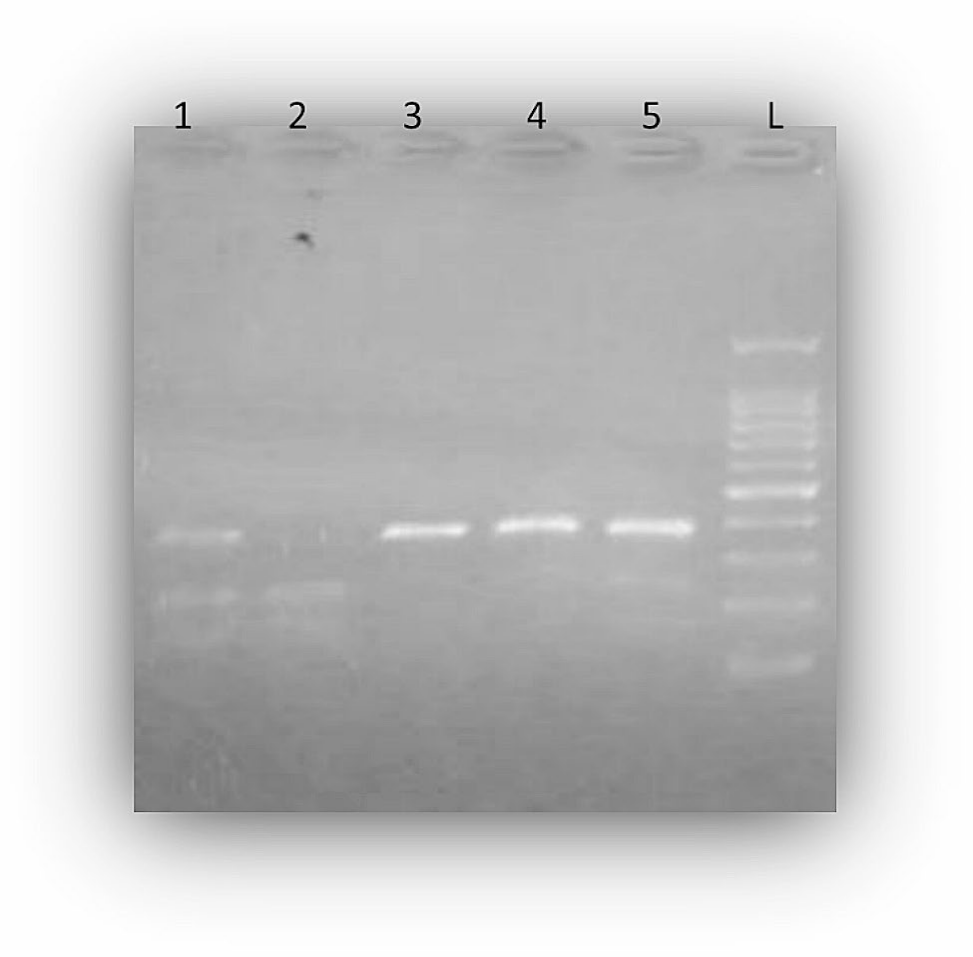
Electrophoresis visualization of the RFLP-PCR products. The genotype TT/TT displayed two bands, 147 bp and 224 bp (well 2). The heterozygous genotype TT/-- exhibited three bands: 224 bp, 147 bp, and 371 bp (wells 1 and 5). The genotype --/-- showed a single band of 371 bp (wells 3 and 4). L is a 100-bp ladder

Then, we evaluated the association between the polymorphism rs10573247 and the risk of breast cancer using RFLP-PCR and electrophoresis (Fig. 4). Table 1 presents a summary of the clinicopathological features of the breast cancer patients.

**Table 1:** Summary of clinicopathological characteristics of breast cancer patients

Condition	Status	Numbers (%)
**Metastasis**	Positive	**36 (25.5)**
	Negative	**42 (30)**
	Unknown	**63 (44.5)**
**Stage**	I	**18 (13)**
	II	**19 (13)**
	III	**24 (17)**
	IV	**14 (10)**
	Unknown	**66 (47)**
**ER**	Positive	**77 (54.6)**
	Negative	**51 (36)**
	Unknown	**13 (9.4)**
**PR**	Positive	**74 (52)**
	Negative	**51 (36)**
	Unknown	**16 (12)**
**HER2**	Positive	**76 (54)**
	Negative	**45 (32)**
	Unknown	**20 (14)**

Among the controls, the observed genotype frequencies of rs10573247 did not deviate significantly from those expected under the Hardy-Weinberg equilibrium (χ2 = 0.29, df = 1, *p* > 0.05). However, among the patients, there was a significant deviation from the Hardy-Weinberg equilibrium (χ2 = 14.64, df = 1, *p* < 0.05).

The results showed that the genotype TT/ delTT (*p* = 0.005) and the genotype delTT/delTT (*p* = 0.029) were associated with an increased risk of developing breast cancer compared to the genotype TT/TT (Table 2).

Furthermore, stratification analysis revealed that the status of ER, HER2, and the grade of patients were not associated with the rs10573247 polymorphism. However, patients with stages 3 and 4, compared to stages 1 and 2, were significantly associated with the genotype delTT/delTT (OR = 12.75, *p* = 0.028, 95% CI = 1.30-124.36). Also, there was a borderline significant association between the genotype TT/ delTT and PR positive compared to PR negative patients (OR = 3.64, *p* = 0.055, 95% CI = 0.97–13.59).

**Table 2:** Genotype and allelic frequencies of the polymorphism rs10573247 in breast cancer patients and the healthy controls

rs10573247	Controls (%)	Patients (%)	OR	95% CI	*P*
Genotype					
TT/TT TT/ delTT delTT/delTT	24 (19.5) 64 (52.0) 35 (28.5)	11 (7.8) 89 (63.1) 41 (29.1)	1 3.034 2.555	- 1.387–6.636 1.098–5.945	- 0.005 0.029
Allele					
TT delTT	112 (45.5) 134 (55.5)	111 (39.3) 171 (60.7)	1 1.28	- 0.91–1.82	- 0.152

## Discussion

The HMGA2 gene plays a role in carcinogenesis [19], and previous studies have shown that HMGA2 mRNA is overexpressed in breast cancer [8, 9]. There are studies about the association of polymorphisms in HMGA2 with the risk of some cancers, but there is no report about HMGA2 polymorphisms in breast cancer.

Previous studies have shown that HMGA2 mRNA is a target of several microRNAs [20–23], thereby polymorphisms located in the 3’ UTR of the HMGA2 gene can lead to alterations of miRNA binding sites that may be linked to cancer risk. Regrading that, in this study, we first did bioinformatic analysis to select the unstudied functional polymorphism located in the 3’-UTR of HMGA2 genes.

Our bioinformatic analysis revealed that deletion polymorphism rs10573247, which is located in the 3’-UTR of HMGA2, may be a binding site for eight miRNAs, and different alleles of this polymorphism could potentially disrupt the binding of two miRNAs, including hsa-miR-3125 and hsa-miR-4476. Interestingly, our results from KEGG analyses showed that HMGA2 is involved in cancer through miRNA.

Then, we performed a case-control study to assess the association between this polymorphism and the risk of breast cancer. Our results showed that the genotypes TT/delTT and delTT/delTT increased the risk of breast cancer.

In line with our results, previous studies have shown that SNP rs6581658 in the HMGA2 gene was associated with the risk of glioma [24] and rs8756 in the 3’ UTR of the HMGA2 gene was associated with the risk of neuroblastoma [25]. Also, rs968697 of the HMGA2 gene polymorphism in the promoter was significantly associated with CRC risk [26] and Wilms tumor [27].

Our results from enrichment analyses also showed that HMGA2 is involved in cellular senescence, DNA repair, differentiation, and chromatin remodeling and is also involved in carcinogenesis. In addition, our results from protein-protein interaction using the STRING database show that HMGA2 interacts with the SMAD family, the IGF2BP family, and the P53 proteins.

Previously, various studies showed expression of the Snail1 gene by binding of HMGA2 and smad3 on its promotor, leading to inhibition of E-cadherin and occludin expression. Also, it was reported that HMGA2 regulates Smad2, Smad3, and TGFβRII, which results in control of TGFβ pathway activation [28–30].

In line with our bioinformatic analyses, it was reported that HMGA2 directly binds to p53 and MDM2 and forms a protein complex with them. Interestingly, one of the involvements of HMGA2 in colorectal cancer [31] and carcinoma of fallopian tubes [32] is MDM2-mediated p53 ubiquitination and degradation.

The IGF2BPs (IGF2 mRNA binding proteins) family contains IGF2BP1, IGF2BP2, and IGF2BP3. HMGA2 with LIN28B and IGF2BP1 form a complex that can mediate the oncogenic process by inhibiting let-7 [33, 34]. Also, HMGA2 can be an oncogenic driver in embryonic rhabdomyosarcoma through the HMGA2-IGFBP2-NRAS axis [35]. Regarding the important role of HMGA2 in critical processes related to cancer and the interaction of it with crucial proteins correlated with cancer, including P53 and the rs10573247 polymorphism in the 3’-UTR of HMGA2, it is reasonable that it is associated with the risk of breast cancer.

The strength of the present study is the bioinformatic analyses that enforced the experimental methods for the role of the rs10573247 polymorphism on breast cancer risk.

In conclusion, our bioinformatics data supports the significance of the 3’ UTR of HMGA2 as a binding site for multiple miRNAs, and our experimental results have shown a significant association between the rs10573247 polymorphism of the HMGA2 gene and breast cancer; however, more research is needed to confirm these associations and further understand the underlying mechanism.

### Electronic supplementary material

Below is the link to the electronic supplementary material.

Supplementary Material 1

## Data Availability

No datasets were generated or analysed during the current study.
