# Supplementary figures and images for: Association of deletion polymorphism rs10573247 in the HMGA2 gene with the risk of breast cancer: bioinformatic and experimental analyses
Source: World J Surg Oncol. 2024 May 28;22:142. doi: 10.1186/s12957-024-03415-4 (PMC11131319; doi:10.1186/s12957-024-03415-4)

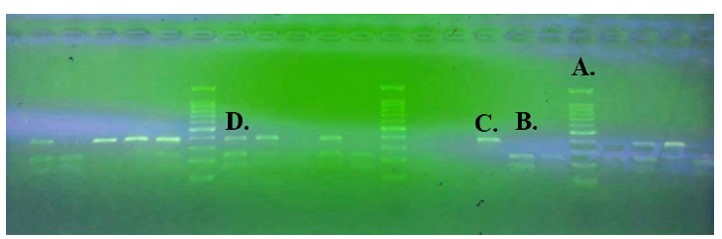

Supplement: Supplementary file 1 — Supplementary Material 1 [file 12957_2024_3415_MOESM1_ESM.jpg]
